# Binding of Protein Factor CTCF within Chicken Genome Alpha-Globin Locus

**Published:** 2016

**Authors:** E. S. Kotova, S. B. Akopov, D. A. Didych, N. V. Petrova, O. V. Iarovaia, S. V. Razin, L. G. Nikolaev

**Affiliations:** Shemyakin-Ovchinnikov Institute of Bioorganic Chemistry, Russian Academy of Sciences, 16/10 Miklukho-Maklaya St., Moscow 117997, Russia; Institute of Gene Biology, Russian Academy of Sciences, 34/5 Vavilov St., Moscow 119334, Russia

**Keywords:** globin genes, transcription factor CTCF, erythroid differentiation

## Abstract

A systematic search for DNA fragments containing potential CTCF transcription factor binding sites in the chicken alpha-globin domain and its flanking regions was performed by means of the two-dimension electrophoretic mobility shift assay. For the alpha-globin domain fragments selected, the occupancy by the CTCF in erythroid and lymphoid chicken cells was tested by chromatin immunoprecipitation. Only one of 13 DNA fragments capable of CTCF binding *in vitro *was efficiently bound to this protein *in vivo *in erythroid cells, and somewhat less efficiently – in lymphoid cells. So, binding of CTCF to the DNA fragment *in vitro *in most cases does not mean that this fragment will be occupied by CTCF in the cell nucleus. Yet, CTCF binding *in vivo*, as a rule, is accompanied by the binding of the protein to this DNA region *in vitro*. During the erythroid differentiation, no significant changes in CTCF binding to the DNA fragments studied were detected.

## INTRODUCTION

In chicken, alpha-globin encoding genes *HBZ*, *HBAD,* and *HBAA *are located in the alpha-globin domain on chromosome 14. The chicken alpha-globin domain belongs to a class of open domains which have certain inherent peculiarities; it is located in a gene-rich region, is sensitive to nucleases in all types of cells, and is replicated in the early S-phase of the cell cycle. The cluster of alpha-globin genes is flanked by housekeeping genes, which are actively transcribed in all studied cell types [1]. The major regulatory element (MRE) of the domain is located approximately 20 kbp upstream from the globin genes [2] and contains an erythroid-specific promoter of whole domain transcript [3]. The enhancer and silencer active in chicken erythroblasts are found near the 3’-end of the *HBAA *gene. In erythroid differentiation, the acetylation status of histone H4 changes in the entire domain [4].

The CTCF transcription factor is thought to participate in various gene regulatory networks, including transcription activation and repression, formation of independently functioning chromatin domains, regulation of imprinting, etc. The fundamental properties of CTCF allow it to act as a transcription factor, an insulator protein, and as a component of boundary elements distributed throughout the genome, which can recruit various factors that appear in response to different internal and external signals [5, 6]. Previously, several CTCF-binding sites were identified in chicken alphaglobin locus. First of all, the M9 and C10–C14 sites located in sequences with insulator functions which bind to CTCF in erythroid and non-erythroid cells [7], and a CTCF-dependent silencer (CDS [8]) which binds to CTCF in HD3 and 6C2 erythroid cells. In addition, the ChIP-seq techique allowed researchers to identify several CTCF-binding sites in the erythrocytes of fiveand ten-day chick embryos (referred to herein as 5d1– 5d3, 10d1–10d3 [9]). One of these sites, 5d1/10d2, may be involved in the switching-on of the globin genes activity in development [10].

In this work, we have undertaken a systematic search for potential CTCF-binding sites in the chicken alpha-globin domain and its flanking regions using a two-dimensional electrophoretic mobility shift assay (2D-EMSA) developed by us earlier [11, 12]. Chromatin immunoprecipitation and real-time PCR analysis were used for further identification of fragments that are occupied by CTCF in erythroid and non-erythroid cells among the selected fragments.

## MATERIALS AND METHODS

**Cell cultures**

The chicken erythroblasts line HD3, transformed by the avian erythroblastosis virus (clone A6, line LSCC, [13]), and the chicken B-lymphoid DT40 cell line (CRL- 2111), were grown in a DMEM/F12 (1:1) medium (Invitrogen) supplemented with 2% chicken and 8% fetal calf serum at 37°C and 5% CO_2_. For DT40 cultivation, the medium was further supplemented with 2-mercaptoethanol to a concentration of 50 μM. Terminal erythroid differentiation of HD3 cells was induced by incubation of the cells for 12 hours in the presence of 20 μM of a iso-H-7 protein kinase inhibitor (1-(5-isoquinolinylsulfonyl)- 3-methylpiperazine dihydrochloride, Sigma-Aldrich) at pH 8.0 and 42°C in 100% air atmosphere as described previously [14]. Benzidine staining was used to control cells differentiation [15]. 1 μL of a 30% H_2_O_2_ solution was added to 25 μl of a 0.4% (w/v) benzidine solution (Sigma) in 4% acetic acid, the resulting solution was mixed with 25 μl of the cell suspension, incubated for 10 min, and a light microscope was used to identify benzidine-positive cells stained with a dark blue color. Hemoglobin-containing (benzidine- positive) cells accounted for 21% of the cells after 12 hours of incubation. Under these conditions, the alpha- globin gene transcriptional level is close to its maximum but continues to increase [16].

**CTCF protein and antibodies**

The full-length chicken CTCF protein, containing a polyhistidine (6 × His) sequence, was synthesized in COS-1 cells and partially purified by the method described previously [17]. Rabbit polyclonal antibodies to a fragment of chicken CTCF (residues 86–233) were prepared according to [17, 18].

**Construction of the alpha-globin locus short fragments library**

DNA of CH_2_61-75C12 clone of bacterial artificial chromosome (BAC, obtained from CHORI BACPAC Resource Center, https://bacpac.chori.org) containing a 227,366 bp chicken alpha-globin locus insert was purified using a Plasmid Midi Kit (Qiagen) and treated with Plasmid-Safe ATP-Dependent DNase (Epicentre) according to the manufacturers’ recommendations.

The library of short fragments was obtained essentially according to [19]. Two BAC DNA samples were digested with either Sau3AI or Csp6I (Fermentas), and ACTGAGGTCGACTATCCATGAACA library primer was attached to the sticky ends. The obtained sub-libraries were amplified by PCR (21–24 cycles) using the same primer and a Encyclo PCR kit (Evrogen) in the presence of 1.5 M betaine and 5% dimethyl sulfoxide as follows: 95°C, 30 sec; 55°C, 30 sec; 72°C, 90 sec. The sublibraries were combined and purified using a QIAquick PCR Purification Kit (Qiagen).

PCR amplification of the M9, CDS, and HBAD fragments with the obtained libraries as templates was performed using an Encyclo PCR kit (Evrogen) in the presence of 1.5 M betaine, and 5% dimethyl sulfoxide. The following pairs of primers were used: TCAGGAAGAAAGAATGGGAAA and CCTGCGTTTTAGCTGATTGG for M9; TCCCAGCACCTCGCAGTGCA and GCACAAGGCTCAAAGGTGAGACA for CDS; CCCAGACCAAGACCTACTTCC and GCTGAGGTTGTCCACGTTCTT for HBAD.

Starting with the 24^th^ PCR cycle, 2.5 μL aliquots were taken from the reaction mixture every three cycles and analyzed in 1% agarose gel.

**Electrophoretic mobility shift assay (EMSA)**

The selected fragments 1–13 were amplified on a plasmid DNA template, isolated from the corresponding clones of the arrayed library, for 10 cycles (94°C, 30 sec; 60°C, 30 sec; 72°C, 90 sec) using the library primer. Next, an aliquot of the reaction mixture was used for PCR radiolabelling according to [12]. For electrophoretic mobility shift assay ~5 ng (30000–50000 cpm) of the labeled DNA fragment were mixed with 1 μg of poly(dI-dC), 1–2 μg (as protein) of a nuclear or cytoplasmic extract or 2 μL of a purified CTCF protein solution in 20 μl of a final volume of 12 mM HEPES-KOH pH 7.9, 12% glycerol, 60 mM KCl, 0.3 mM EDTA, and 0.6 mM DTT. 4.5 ug of anti-CTCF antibodies or 3 μg of monoclonal antibodies to poly-histidine (Sigma, H1029) were added for the supershift assay. The mixture was incubated for 20 min at room temperature, resolved in 5–7.5% polyacrylamide gel prepared with a 50 mM Tris-borate buffer, pH 8.3, 0.5 mM EDTA, and autoradiographed for 16–40 hours.

A two-dimensional electrophoretic mobility shift assay (2D-EMSA) was performed as described previously [12] with minor modifications. PCR amplification was done in the presence of 1.5 M betaine and 5% dimethyl sulfoxide using the Encyclo PCR kit (Evrogen). 10 μL of the protein fraction containing ca. 0.5 pmol CTCF was used for the first round of two-dimensional EMSA, and 1 μL of the same fraction was used for the second round. The resulting library of CTCF-binding DNA fragments was cloned into pGEM-T plasmid (Promega) and arrayed in 96-well plates. A total of 230 clones were sequenced and mapped on the Gallus gallus genome (galGal4).

**Chromatin immunoprecipitation (ChIP)**

Chromatin immunoprecipitation was performed according to the previously described method [20]. Approximately 3×10^7^ exponentially growing (for DT40 and HD3) or collected 12 hours after the initiation of induction (for induced HD3) were fixed with 1% (v) of formaldehyde in 60 mL of a DMEM/F12 medium (1:1) for 8 min. The cells were pelleted by centrifugation for 4 min at 700 *g *and 4°C, washed with PBS, containing 1 mM AEBSF and a 1 μL/mL protease inhibitor cocktail (Sigma, P8340), re-pelleted, re-suspended in 200 μL of 50 mM Tris-HCl pH 8.0, 1% SDS, 10 mM EDTA and incubated for 10 min on ice for lysis. The cells were then sonicated using a Cole-Parmer CP750 processor (30% amplitude, 30 3-sec cycles with 10-sec intervals). Cell debris were removed in a microcentrifuge (10 min, 13,000 rpm, 4°C), the supernatant was diluted 10 times with 16.7 mM Tris-HCl pH 8.0, 16.7 mM NaCl, 1.2 mM EDTA, 1% Triton X-100, 0.01% SDS, 1 mM PMSF and the 1 μL/mL protease inhibitor cocktail. At this stage, an input control aliquot was withdrawn. Cell lysate was purified from nonspecifically bound proteins by pre-incubation with protein-A-agarose (Invitrogen) and then incubated with 2 μg of polyclonal antibodies to CTCF or control rabbit polyclonal antibodies to thaumatin (kindly provided by E.A. Stukacheva) overnight at 4°C and constant stirring. DNA-protein complexes were collected on protein-A-agarose, washed and eluted from the vehicle with elution buffer (1% SDS, 0.1 M NaHCO_3_, 2 x 15 min) at room temperature. NaCl was added to the solution to a concentration of 0.2 M, followed by RNase A and proteinase K, and the mixture was incubated at 65°C for 4 hours to reverse the crosslinks. DNA was extracted twice with a phenol-chloroform mixture and precipitated with ethanol overnight at 4°C in the presence of 20 μg glycogen as a carrier. The DNA fragments were collected by centrifugation, dissolved in water, and analyzed using quantitative real- time PCR on a MX3000P thermocycler (Stratagene) and qPCRmix-HS SYBR reaction mixture (“Evrogen”) in a volume of 25 μl for 40 cycles: 95°C, 30 sec; 61–65°C (for different primers), 30 sec; and 72°C, 60 sec. The efficiency of PCR was calculated using the LinRegPCR software [21].

## EXPERIMENTAL

A fragment of a chicken lysozyme gene F1 silencer [22] and a fragment of the promoter region of the chicken *MYC *gene [23] were used as positive controls for quantitative PCR. A CTCF non-binding enhancer fragment from the chicken beta-globin locus [8] and a fragment of the alpha*-D*-globin (*HBAD*) exon gene were used as negative controls. DNA fragments were amplified on the chicken genomic DNA template using the following primers: CAGCACAGTTCTGGCTATGAAA and CCTCAGCTGGGGTCAATAAGT (lysozyme gene silencer); AAGCAGCGAGGAGCGCCCTTT and TACTACAAGGAGAGGTCGGAAGT (*MYC *gene promoter); GGGCAGGTTGCAGATAAACA and TAACCCCCTCTCTTCCCTCA (enhancer from beta-globin locus); CCCAGACCAAGACCTACTTCC and GCTGAGGTTGTCCACGTTCTT (*HBAD* gene exon); TGTGGTCATCCATGTCCTCAATC and GGAAGCTTTTTGCCAAGGAGAA for 10d1; GCTCTTCCTCACCCAGGTTTCT and CATCCAGCCCTCTCCAAACA (10d2, 8, and 5d1); TGACCCATCTTGCAATGGATACT and GTTTGGGAACTCTCTCTCCATCC (10d3); ATAGGACTTCCCTGCTTCCATCT and GTTGGAGTGTTGTGGTCTTCTCC (5d2); GTGAGGAGAGGGCGAAGTTTATT and GCTCCCTGAGCTCCTCACCT (5d3); ATAACTTGGCACGCAAACTAGCA and TTTGGAAAGTGCTGTGGGTAAAG (fragment 1); TTCTACACTTGTCCCTCCTTTTCA and CCTATTTTGTGGCTGCATTCTTC ( f r a g - ment 2 ) ; GGAGCTCAGCAGGCAGAAACTA and GCTAAGGCAAAGGCTCTGTTGT (fragment 3 ) ; CTCTGCATTGCTGTGTGTGTTTT and ATGGTGGTTATCTCAGGGGTTTT (fragment 4); GGTACGTTCTCAGTGCCCAAAC and CCACCTGCAGACCTAACCTGTC ( f r a g - ment 5); CAGCTCTTCTGGCTCATTTGTCT and ATCTCCCTTTCAGTCCCCTTCTC (fragment 6); TTTCACCCCAGAAGTTCATGCT and CCCAGTGTGGAAGCCATTTATC (fragment 7); CATGGGCAGCAAACACACAG and TCCATTTCCAGCGGTTCTTATC (fragment 9); AGGTAGGACTCAGCAGGGACAG and GGGACAAGTAGCTGGGACAAAA (fragment 10); CTGGAGATACCCATGGCAGAAC and TTTGTGGCCAACGTCAAACTAC (fragment 11); GGTTTGCCTTTCTTGCTCTG and ATGCCCATCTCACTTGCTCT (fragment 12); CGTACCAGCACCAGACAAACAG and TCGACTGTTGAAGGAGGCATAA (fragment 13).

Data were analyzed using genome browser resources (UCSC Genome Browser, http://genome.ucsc.edu) [24] and NCBI-BLAST (http://blast.ncbi.nlm.nih.gov/Blast. cgi).

## RESULTS AND DISCUSSION

**Selection of CTCF-binding sequences using 2D-EMSA**

**Fig. 1:**
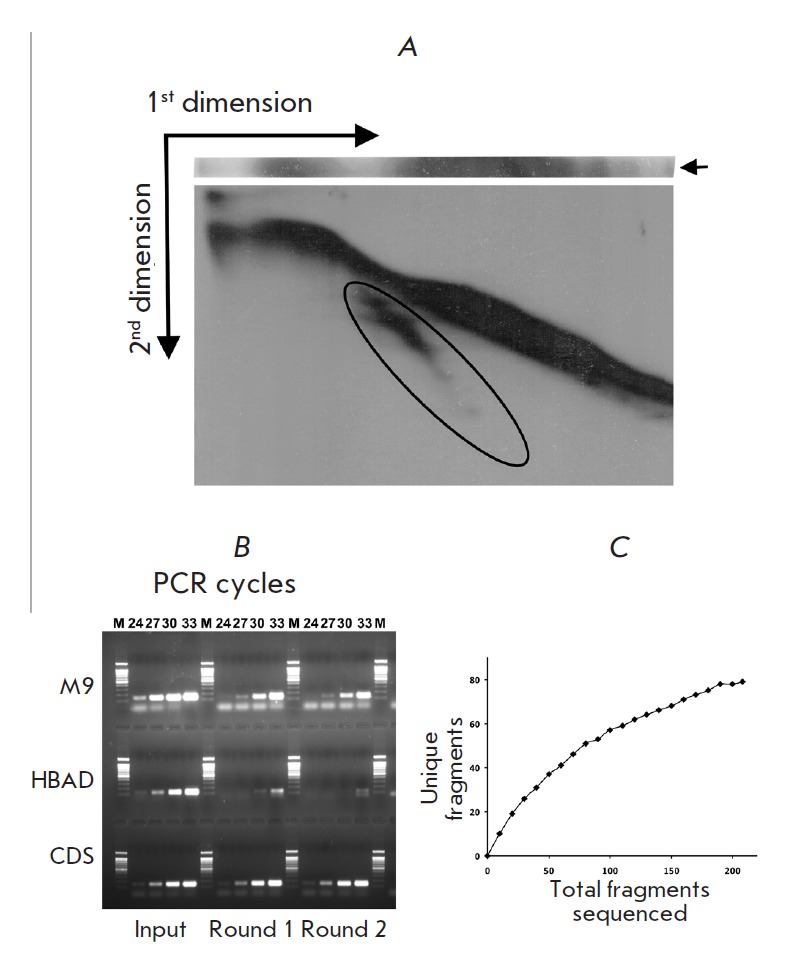
Preparation and characterization of the library of CTCF-binding fragments. (A) Selection of CTCF-binding fragments by means of the two-dimensional electrophoretic mobility shift assay (2D-EMSA). The results of two-dimensional electrophoresis for the second selection round are shown. Region containing selected CTCF-binding fragments is outlined by the oval. For detail, see text. (B) Estimation of the degree of enrichment with the CTCF-binding fragments for the library obtained. Initial DNA and DNA after first and second 2D-EMSA selection rounds were used as a template for PCR with primers targeted to CTCF-binding sequences from the chicken alpha-globin locus: CDS (CTCF-dependent silencer) and M9 sequence. Sequence from *HBAD *gene exon which does not bind CTCF was used as a negative control. (C) Rarefaction curve obtained during sequencing of the CTCF-binding fragments library

To obtain libraries of CTCF-binding sequences by two-dimensional EMSA (2D-EMSA, [12]), the artificial bacterial chromosome (BAC) containing a 227366 bp insert, which overlaps the chicken alpha-globin locus and includes extensive flanking regions, was digested to completion with either the Sau3AI or Csp6I restriction enzyme. Synthetic adapters were attached to the resulting sticky ends, amplified by PCR, and both hydrolysates were mixed in equal proportions. The resulting library of short fragments (approximately 1,000 fragments with an average length of ca. 500 bp) was ^32^P-labeled and mixed with a protein fraction enriched in full-length CTCF, expressed in COS-1 cells [17]. The reaction mixture was then electrophoretically separated by non-denaturing polyacrylamide gel (first dimension). The region with the sample was cut out, incubated in SDS-containing buffer to disrupt the DNA-protein complexes, and the DNA fragments were separated in SDS-containing gel (second dimension). The region containing the most fragments originally bound to CTCF (outlined by the oval in *Fig. 1A*) was cut out from the gel and the DNA fragments were eluted and amplified. The procedure was repeated to improve the efficiency of selection.

The specificity of selection was checked by amplification of the resulting and the original libraries with primers to chicken alpha-globin locus sequences, which bind to CTCF according to the published data: namely, CDS (CTCF-dependent silencer) [8] and the M9 sequence [7]. The sequence of *HBAD *exon which does not bind to CTCF was used as a negative control. The results of amplification are shown in *Fig. 1B*.

As can be seen from *Fig. 1*, after two rounds of selection PCR products of the CDS and M9 regions become visible after 24 and 27 cycles of amplification, respectively, while the product of the control *HBAD* gene fragment that does not bind to CTCF becomes visible only after 33 cycles. Since all three fragments are amplified from the original library with approximately equal efficiency (see the input lane in *Fig. 1B*) a rough estimation of the degree of enrichment with the CTCFbinding fragments for the library obtained is ~64–512 times.

The DNA fragments obtained after the second round of the selection were cloned into a pGEM-T vector, white colonies (230) were arrayed in 96-well plates, and their inserts were sequenced. Among these sequences, 22 corresponded to fragments of BAC, *Escherichia coli* genomic DNA or chimeric fragments, and 208 belonged to the alpha-globin locus. 79 unique sequences were identified. The constructed rarefaction curve (*Fig. 1B*) indicates that the sequencing was performed with a depth sufficient to identify most of the potential CTCFbinding fragments of the locus.

**Fig. 2:**
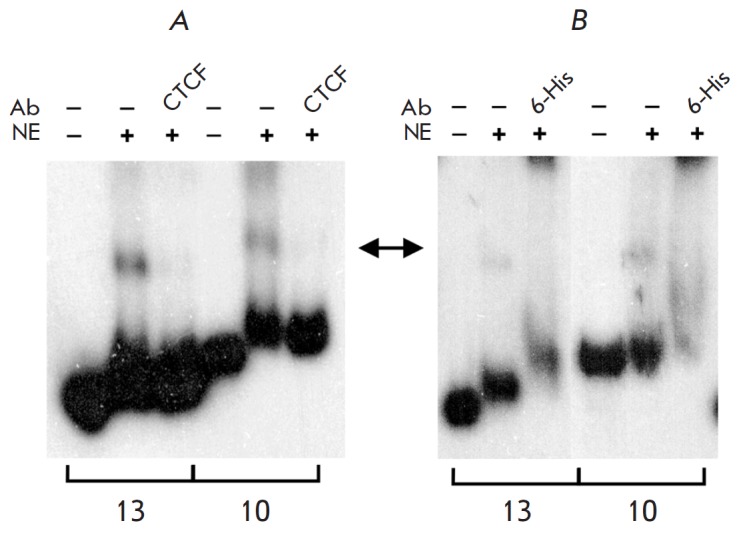
CTCF binding to selected DNA fragments 10 and 13. Anti-CTCF (A) and anti-polyHistidine (B) antibodies were used for the supershift assay. AB – antibodies, NE – nuclear extract

Ten selected DNA fragments (1–4, 6–10, 13) were used as probes to test their ability to bind CTCF by electrophoretic mobility shift and supershift assays (EMSA, supershift). Two fragments (10 and 13) are shown in *Fig. 2*. All 10 fragments were able to bind CTCF, which indicates the high efficiency of the selection.

**Distribution of potential CTCF binding sites**

**Fig. 3:**
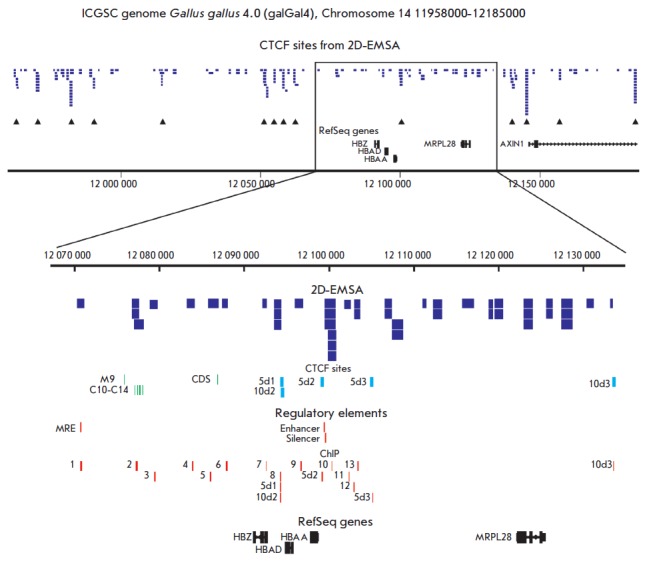
Distribution of CTCF binding sites and some regulatory elements in the region overlapping the chicken genome alpha-globin domain. Upper map shows the positions of all selected DNA fragments. The arrows indicate DNA regions with high affinity to CTCF. Lower part shows the enlarged map of the immediate surroundings of the globin genes. In the “CTCF sites” panel the identified previously CTCF binding sites M9, C10-C14 [7], CDS [8] and 5d1-5d3, 10d1-10d3 [9] are shown, the “Regulatory elements” panel demonstrates the positions of the MRE [2] and the enhancer and silencer [25]. In the ChIP region the positions of DNA fragments amplified in the chromatin immunoprecipitation experiment are shown (see text)

All 208 sequenced fragments were mapped to the Gallus gallus genome (galGal4, 2011). A table with the coordinates of all mapped DNA fragments in BED format is available upon request. A full map of the fragments distribution is presented in the upper part of *Fig. 3*. As can be seen, the locus had a number of sites with higher selection efficiency (indicated by vertical arrows); i.e., with higher affinity for CTCF in EMSA conditions. The bottom part of *Fig. 3* shows an enlarged map of the immediate surroundings of the globin genes with indicated genes positions (RefSeq), as well as some previously identified regulatory elements, in particular the enhancer/silencer [25] and MRE (Major Regulatory Element, [2]). It also shows DNA fragments that had been previously identified in various cell types and tissues as capable of binding CTCF: M9, C10-C14 [7], and a fragment of the CTCF-dependent silencer [8]. CTCF-binding fragments 5d1–5d3 and 10d1–10d3 have been previously identified by ChIP-seq in five- and ten-day chick embryos, respectively [9].

As can be seen from *Fig. 3*, the vast majority of previously identified CTCF binding sites are located in or very close to the regions of high selection efficiency; i.e., strong CTCF-binding in EMSA conditions. The binding site 10d1, located outside the enlarged section of the map, is also located in the area with high affinity to CTCF. It should be noted that the binding site and the cross-linking position in chromatin immunoprecipitation may not match exactly due to DNA bending [26, 27]; i.e., fragments identified by EMSA and ChIP do not necessarily overlap, even though they should be located close to one another.

**CTCF binding *in vitro* and *in vivo* in the region of alpha-globin genes**

**Fig. 4:**
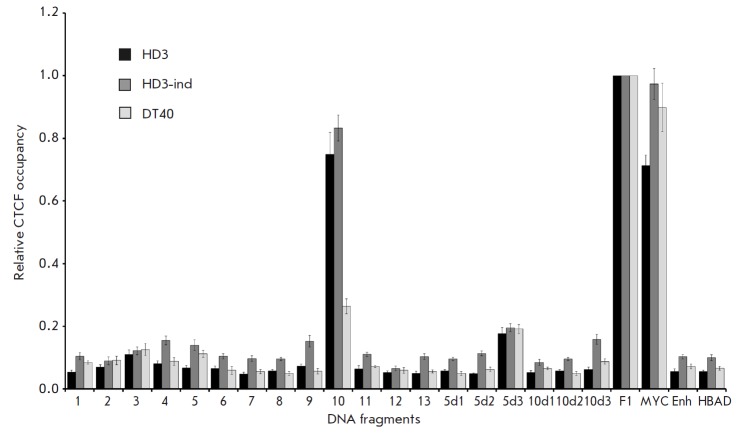
CTCF binding to DNA regions *in vivo *as revealed by chromatin immunoprecipitation and a real-time PCR analysis. The results for HD3 cells, HD3 induced to erythroid differentiation, and for B-lymphoid DT40 cells are presented. Primers were targeted to the DNA fragments selected in this work (1-13) and to six fragments identified in [9] (5d1-5d3, 10d1- 10d3). F1, MYC – positive controls; Enh, HBAD – negative controls. The data are normalized to binding CTCF with the F1 fragment. Error bars indicate the standard errors of the mean.

To compare the CTCF binding to DNA in a living cell and detected by EMSA, we performed chromatin immunoprecipitation for 13 DNA fragments from the globin region, as well as for the 5d1–5d3 and 10d1–10d3 fragments [9] in three cell types: HD3 cells, HD3 induced to erythroid differentiation, and B-lymphoid DT40. The positions of DNA fragments amplified during chromatin immunoprecipitation are shown in *Fig. 3* (ChIP panel), and the results of immunoprecipitation are presented in *Fig. 4*.

*Fig. 4* demonstrates that fragment 10, located near the 3’-end of *HBAA*, is the only one to display a high degree of occupancy by CTCF, close to that observed for the positive controls (F1, MYC). A high degree of CTCF binding is observed in HD3 cells and induced HD3 cells, while CTCF binding to this site in DT40 cells is significantly lower. Remarkably, the position of fragment 10 coincides with the position of the genomic region fragment with the strongest CTCF binding *in vitro *(*Fig. 3*).

In addition to fragment 10, another fragment to stand out is 5d3, whose CTCF occupancy is reliably above the negative control level for all three cell types but is substantially lower than that of fragment 10 in HD3 and HD3-ind cells. Some excess over the negative control is observed also for fragments 4, 5, 9, and 10d3 in HD3-ind cells only, but the extent of this excess is small and does not allow us to claim with certainty that these fragments bind CTCF.

Thus, most DNA fragments (17 out of 18) that bind to the purified CTCF protein in EMSA conditions are not occupied by CTCF in the cell nucleus of the studied cell types. This fact can be attributed to the following reasons:

1. Methylation of cytosine in CpG dinucleotides disrupts its binding to CTCF [28, 29]. However, only about 30% of CTCF binding sites contain the CpG sequence [30]: therefore, DNA methylation at the CTCF site can only partially explain the results.

2. CTCF binding is limited to sites with a suitable structure of chromatin/histone modifications and/or presence of other transcription factors nearby that facilitate CTCF binding [31].

Most likely, both reasons play a role in limiting CTCF binding [32].

Obviously, some of the sites that were not occupied by CTCF in our chromatin immunoprecipitation experiments (*Fig. 4*) can bind this protein in other types of cells and tissues. For example, the DNA fragments 5d1, 5d2, 10d1–10d3, which do not bind CTCF in DT40 and HD3 cells (*Fig. 4*), bind to it in chick embryo erythroblasts [9].

The 5d3 fragment is a special case. It binds CTCF according to the results of chromatin immunoprecipitation (*Fig. 4*) and according to [9], but it does not overlap with any of the selected fragments. The CTCF-binding M9 fragment behaves similarly [7], but its presence in the library is confirmed by PCR (*Fig. 1B*). Perhaps both of these DNA fragments did not fall into the sequenced pool.

## CONCLUSIONS

On the basis of these experiments we can conclude that there is a unilateral relationship between the CTCF-binding efficiency of a fragment under EMSA conditions (*in vitro*) and its degree of occupancy by a CTCF protein in ChIP conditions. Binding of CTCF to a DNA fragment *in vitro *in most cases does not mean that this fragment will be occupied by CTCF in the cell nucleus. In contrast, CTCF binding *in vivo*, as a rule, is accompanied by the binding of the protein to this DNA region *in vitro*. Furthermore, these results show that erythroid differentiation has no significant impact on the CTCF binding of the studied DNA fragments.

The only site which strongly binds CTCF in erythroid cells, HD3 and HD3-ind, binds this protein in lymphoid DT40 cells with significantly (2–3 times) weaker efficiency; i.e., CTCF binding to this site is distinctly tissue-specific. At the same time, there are no significant differences in CTCF binding in a HD3 erythroblast cell line and in cells of the same line stimulated to erythroid differentiation.

## Abbreviations

EMSA: Electrophoretic Mobility Shift Assay
PBS: Phosphate Buffered Saline
AEBSF: 4-(2-AminoEthyl) BenzeneSulfonyl Fluoride
ChIP: Chromatin ImmunoPrecipitation
ChIP-seq: Chromatin ImmunoPrecipitation with the subsequent mass sequencing
